# Failure in post-transcriptional processing is a possible inactivation mechanism of AP-2 **α** in cutaneous melanoma

**DOI:** 10.1054/bjoc.2000.1145

**Published:** 2000-05-18

**Authors:** J M Karjalainen, J K Kellokoski, A J Mannermaa, H E Kujala, K I Moisio, P J Mitchell, M J Eskelinen, E M Alhava, V M Kosma

**Affiliations:** Departments of Surgery, Clinical Pathology, Oral and Maxillofacial Unit, Oncology, Chromosome and DNA Laboratory, Pathology and Forensic Medicine, University of Kuopio and Kuopio University Hospital, Kuopio, Finland; Department of Biochemistry and Molecular Biology, Pennsylvania State University, 332 South Frear, University Park, PA, 16802-4500, USA

**Keywords:** cutaneous melanoma, gene expression, AP-2, mRNA in situ hybridization, loss of heterozygosity

## Abstract

The loss of transcription factor AP-2α expression has been shown to associate with tumourigenicity of melanoma cell lines and poor prognosis in primary cutaneous melanoma. Altogether these findings suggest that the gene encoding AP-2α (TFAP2A) acts as a tumour suppressor in melanoma. To learn more of AP-2α’s down-regulation mechanisms, we compared the immunohistochemical AP-2α protein expression patterns with the corresponding mRNA expression detected by in situ hybridization in 52 primary melanomas. Of the 25 samples with AP-2α protein negative areas, 16 (64%) expressed mRNA throughout the consecutive section. Nine specimens (36%) contained equally mRNA- and protein-negative areas, suggesting that the loss of AP-2α protein associated with lack of the mRNA transcript. The highly AP-2α protein-positive tumours (*n* = 27) were concordantly mRNA positive in 25 (92.6%) cases. Thirteen primary tumours were further analysed using microsatellite markers D6S470 and D6S263 for loss of heterozygosity (LOH) of a locus harbouring TFAP2A. LOHs or chromosome 6 monosomy were found in four out of five (80%) informative AP-2α mRNA- and protein-negative tumour areas, but also within five out of 13 (38%) informative AP-2α mRNA-positive tumour areas. This chromosome region is thus suggestive of harbouring a putative tumour suppressor gene of cutaneous melanoma, but this referring specifically to TFAP2A could not be completely verified in this analysis. We conclude that a failure in post-transcriptional processing of AP-2α is a possible inactivation mechanism of AP-2α in cutaneous melanoma. Copyright 2000 Cancer Research Campaign© 2000 Cancer Research Campaign


*British Journal of Cancer* (2000) **82**(12), 2015–2021
© 2000 Cancer Research Campaign
doi: 10.1054/ bjoc.2000.1145, available online at http://www.idealibrary.com on IDEAL®

### Failure in post-transcriptional processing is a possible inactivation mechanism of AP-2α in cutaneous melanoma

**JM Karjalainen[1,6], JK Kellokoski[3,4,6], AJ Mannermaa[5], HE Kujala[5], KI Moisio[6], PJ Mitchell[7], MJ Eskelinen[1], EM Alhava[1] and VM Kosma[2,6]**

Departments of [1]Surgery, [2]Clinical Pathology, [3]Oral and Maxillofacial Unit, Otolaryngology, [4]Oncology, [5]Chromosome and DNA Laboratory, and [6]Pathology and Forensic Medicine, University of Kuopio and Kuopio University Hospital, Kuopio, Finland; [7]Department of Biochemistry and Molecular Biology, 332 South Frear, Pennsylvania State University, University Park, PA 16802-4500, USA

**Summary** The loss of transcription factor AP-2α expression has been shown to associate with tumourigenicity of melanoma cell lines and poor prognosis in primary cutaneous melanoma. Altogether these findings suggest that the gene encoding AP-2α (TFAP2A) acts as a tumour suppressor in melanoma. To learn more of AP-2α's down-regulation mechanisms, we compared the immunohistochemical AP-2α protein expression patterns with the corresponding mRNA expression detected by in situ hybridization in 52 primary melanomas. Of the 25 samples with AP-2α protein negative areas, 16 (64%) expressed mRNA throughout the consecutive section. Nine specimens (36%) contained equally mRNA- and protein-negative areas, suggesting that the loss of AP-2α protein associated with lack of the mRNA transcript. The highly AP-2α protein-positive tumours ($n$ = 27) were concordantly mRNA positive in 25 (92.6%) cases. Thirteen primary tumours were further analysed using microsatellite markers D6S470 and D6S263 for loss of heterozygosity (LOH) of a locus harbouring TFAP2A. LOHs or chromosome 6 monosomy were found in four out of five (80%) informative AP-2α mRNA- and protein-negative tumour areas, but also within five out of 13 (38%) informative AP-2α mRNA-positive tumour areas. This chromosome region is thus suggestive of harbouring a putative tumour suppressor gene of cutaneous melanoma, but this referring specifically to TFAP2A could not be completely verified in this analysis. We conclude that a failure in post-transcriptional processing of AP-2α is a possible inactivation mechanism of AP-2α in cutaneous melanoma. © 2000 Cancer Research Campaign

**Keywords:** cutaneous melanoma; gene expression; AP-2; mRNA in situ hybridization; loss of heterozygosity

The 52-kDa activator protein (AP)-2 is a DNA-binding transcription factor (Mitchell et al, 1987) which has been shown to control transcription of several genes that are related to embryogenesis (Mitchell et al, 1991), cancer-growth suppression or progression (Bosher et al, 1995; Bar-Eli, 1997; Zeng et al, 1997; Turner et al, 1998). To date, three human AP-2-related genes, encoding AP-2α (TFAP2A), AP-2β (TFAP2B) and AP-2γ (TFAP2C) have been cloned (Williamson et al, 1996).

There is increasing evidence that normal function of AP-2α is needed in preventing the tumourigenicity and progression of cutaneous melanoma. The expression of AP-2 is lost in malignant melanoma cell lines (Bar-Eli, 1997). In addition, restored AP-2α expression inhibits growth and metastatic capacity of melanoma cell lines through activation of proto-oncogene/tyrosine kinase receptor c-KIT and down-regulation of MCAM/MUC18 (Bar-Eli, 1997; Huang et al, 1998; Jean et al, 1998). We recently demonstrated in a representative clinical cutaneous melanoma material that loss of AP-2α protein expression is associated with tumour progression and independently predicts poor disease outcome (Karjalainen et al, 1998). Altogether, these findings suggest that the gene encoding AP-2α (TFAP2A) may be a tumour suppressor in primary cutaneous melanoma.

In cutaneous melanomas genetic analyses have preferably concentrated on cell lines and metastases due to the reduced availability of primary tumour material. To date it is not known how the expression of AP-2α protein is down-regulated in primary cutaneous melanoma, and what are the critical steps involved in this process at molecular level.

These issues are extremily important because the knowledge of the AP-2α's regulatory mechanisms in primary cutaneous melanoma specimens will increase our understanding on the melanocytic tumour progression and may thus open new therapeutic options in future. Therefore, we have now compared the immunohistochemically detected AP-2α protein levels with the corresponding AP-2α mRNA expression in consecutive tumour areas of 52 primary cutaneous melanomas derived from our previous larger clinical material (Karjalainen et al, 1998). In addition, we have studied whether loss of heterozygosity (LOH) at the chromosomal location (6p24.3) (Gaynor et al, 1991) of AP-2α gene is the mechanism responsible for the down-regulation of AP-2α. The role of other AP-2 family members was also studied to evaluate the importance of AP-2α in the malignant progression of cutaneous melanoma.

*Received 28 September 1999*
*Revised 13 January 2000*
*Accepted 18 January 2000*

*Correspondence to:* JM Karjalainen, Department of Pathology and Forensic Medicine, University of Kuopio, PO Box 1627, FIN-70211 Kuopio, Finland

### MATERIALS AND METHODS

### Tumour material

The material of this study was derived from a large ($n$ = 273) earlier series of clinical stage I cutaneous melanoma patients, diagnosed and treated in the district of Kuopio University Hospital between 1974 and 1989, with clinicopathological and follow-up data available (Karjalainen et al, 1998). The starting point for the current study was to statistically determine a cut-off level (< 75% vs ≥ 75% of AP-2α-positive cancer cells) that efficiently divided the original ($n$ = 273) series into low-risk (high expression, $n$ = 169, 62%) and high-risk (low expression, $n$ = 104, 38%) groups (other data not shown). Based on this cut-off level, 25 high-risk tumours with low AP-2α expression and 27 low-risk tumours with high AP-2α expression were selected from the original cohort for mRNA in situ hybridization (ISH) analyses (Table 1). As we

**Table 1** The AP-2α protein and mRNA expressions, LOH status of TFAP2A and clinicopathological features of the primary tumours

| Case number | AP-2α protein expression[a] | mRNA/ protein-concordance[b] | LOH[c] (analysed area) | Breslow class[d] | Disease recurrence | Cause of death |
|---|---|---|---|---|---|---|
| 1 | high | +/+ | | 4 | no | alive |
| 2 | high | +/+ | | 3 | no | alive |
| 3 | high | +/+ | yes (mRNA+) | 3 | no | other |
| 4 | high | +/+ | | 3 | no | alive |
| 5 | low | +/– | | 2 | yes | melanoma |
| 6 | high | +/+ | no (mRNA+) | 3 | yes | melanoma |
| 7 | high | +/+ | | 2 | no | alive |
| 8 | low | +/– | | 2 | no | other |
| 9 | high | +/+ | yes[e] (mRNA+) | 4 | yes | melanoma |
| 10 | low | +/– | | 3 | yes | melanoma |
| 11 | high | +/+ | | 3 | yes | other |
| 12 | high | –/+ | | 4 | no | other |
| 13 | low | good | homozygous | 3 | yes | melanoma |
| 14 | low | good | | 4 | yes | melanoma |
| 15 | low | good | no (mRNA+) | 3 | no | alive |
| 16 | low | good | | 2 | yes | melanoma |
| 17 | high | +/+ | no (mRNA+) | 4 | yes | melanoma |
| 18 | low | good | yes[e,f] | 2 | yes | alive |
| 19 | high | +/+ | | 2 | no | alive |
| 20 | low | good | yes (mRNA–) | 3 | no | alive |
| 21 | high | +/+ | | 2 | no | alive |
| 22 | low | +/– | homozygous | 4 | yes | other |
| 23 | high | +/+ | | 3 | no | alive |
| 24 | high | +/+ | | 3 | no | alive |
| 25 | high | +/+ | | 1 | no | other |
| 26 | low | +/– | no (mRNA+) | 4 | no | alive |
| 27 | low | +/– | | 3 | yes | melanoma |
| 28 | low | +/– | no (mRNA+) | 3 | yes | melanoma |
| 29 | low | +/– | | 2 | yes | melanoma |
| 30 | high | +/+ | | 4 | no | alive |
| 31 | low | +/– | | 4 | yes | melanoma |
| 32 | high | +/+ | | 2 | yes | alive |
| 33 | high | +/+ | | 1 | yes | melanoma |
| 34 | high | +/+ | | 2 | no | alive |
| 35 | high | +/+ | | 2 | yes | other |
| 36 | high | +/+ | yes (mRNA+) | 3 | yes | melanoma |
| 37 | low | +/– | | 4 | yes | melanoma |
| 38 | low | +/– | homozygous | 3 | yes | other |
| 39 | high | –/+ | | 4 | yes | other |
| 40 | high | good | no[f] | 4 | yes | alive |
| 41 | low | good | yes[g,f] | 4 | yes | melanoma |
| 42 | high | +/+ | | 4 | yes | melanoma |
| 43 | low | +/– | | 4 | yes | melanoma |
| 44 | low | good | yes[g] (mRNA–) | 4 | no | alive |
| 45 | high | +/+ | | 2 | no | alive |
| 46 | high | +/+ | | 2 | no | alive |
| 47 | high | +/+ | | 2 | no | alive |
| 48 | low | good | homozygous | 4 | no | other |
| 49 | low | +/– | | 3 | yes | melanoma |
| 50 | low | +/– | | 4 | yes | alive |
| 51 | low | +/– | | 4 | no | alive |
| 52 | low | +/– | | 3 | no | alive |

[a]High: ≥ 75% of AP-2α protein-positive cells; low: < 75% of AP-2α protein-positive cells; [b]+/+: consecutive sections are completely mRNA and protein-positive; +/–: corresponding mRNA-positive and protein-negative tumour areas; –/+: corresponding mRNA-negative and protein-positive tumour areas; good: concordant mRNA and protein expression patterns. [c]LOH: loss of heterozygosity. [d]Breslow class: 1: ≤ 0.75 mm; 2: 0.76–1.50 mm; 3: 1.51–4.0 mm; 4: more than 4.0 mm. [e]Monosomy observed. [f]The same result in mRNA-positive as well as in protein and mRNA-negative areas. [g]Monosomy not excluded.

*© 2000 Cancer Research Campaign*

additionally wanted to characterize the AP-2α protein and mRNA expression levels as related to melanocytic tumour progression, seven subsequent metastases of primary melanomas were also studied.

### Histology and immunohistochemistry

The histological and immunohistochemical (IHC) methods have been described in detail previously (Karjalainen et al, 1998). In brief, a rabbit polyclonal AP-2α antibody (C-18) (Santa Cruz Biotechnology, CA, USA), raised against a peptide corresponding to amino acids 420–437 mapping at the carboxy terminus of AP-2 of human origin (Mitchell et al, 1991), was used at a working dilution of 1:2500. The IHC method included antigen unmasking (microwave boiling 2 × 5 min) and standard ABC technique (Vectastain Rabbit ABC Elite Kit, Vector Laboratories, Burlingame, CA, USA) to localize the bound antibody. Constantly positive epidermal and inflammatory cells served as excellent positive internal controls. Melanoma specimens known to be positive for AP-2α were used as additional positive controls. In each batch, a primary melanoma specimen incubated without primary antibody was used as a negative control. Eighteen tumours were stained using the same staining protocol with human AP-2β and AP-2γ specific antibodies (C-20 and T-17, Santa Cruz Biotechnology, CA, USA) at working dilutions of 1:200.

### mRNA in situ hybridization

Deparaffinized 5-μm sections were microwaved in 0.01 M citrate buffer (pH 6.0) for 5 min. The samples were fixed in 4% paraformaldehyde for 20 min, rinsed, pretreated with 0.1 M hydrochloric acid (HCl) and phosphate-buffered saline (PBS), followed by digestion for 20 min with proteinase K (Boehringer Mannheim, Germany) at the concentration of 20 μg $ml^{-1}$. After a short fixation by 4% paraformaldehyde, the sections were rinsed in PBS and in 0.85% sodium chloride (NaCl), dehydrated in graded ammonium acetate alcohols and air-dryed. All specimens were then prehybridized for 2 h at 62°C. Recombinant plasmid pHAP2-Hsma containing human AP-2α exon 2 (transactivation domain) in a pBluescript KS+-vector was linearized to generate antisense- and sense-digoxigenin-labelled probes by DIG RNA labelling Kit (Boehringer Mannheim, Germany). The AP-2α exon 2 region selected for this study is relatively poorly conserved with short amino acid motifs only, and at the RNA level the homology is minimal within the other AP-2 family members. Under the conditions used in this study the probe is thus very unlikely to cross-hybridize to anything else but AP-2α RNA. Probes were added to the hybridization buffer at 5 ng $\mu l^{-1}$ and the sections were hybridized overnight at 62°C in a humified chamber. Subsequently, the sections were washed with increasing stringency. Non-specific binding was blocked with 2% normal sheep serum (Sigma, UK) in 0.1% Triton X/TBS-buffer. The hybridized probe was detected by incubation in anti-digoxigenin alkaline phosphatase conjugate (Boehringer Mannheim, Germany) followed by NBT/BCIP. The reaction was stopped with diluted water, sections were dehydrated and coverslipped with Mountex (Histolab Production AB, Sweden). Consecutive sections of each specimen, stained using the sense probe, served as negative controls and remained negative throughout the series (Figure 1E).

### Microdissection

Twenty-five paraffin-embedded samples were microdissected to be used for the LOH analyses. The microdissection was made using a 24 G needle under a light microscope in carefully matched tumour areas containing at least 70% of tumour cells. Normal tissue was obtained from the same preparates. From each specimen, five 20-μm-thick consecutive sections were subjected to DNA extraction. Whenever possible, the tumour areas that displayed no protein and mRNA signal for AP-2α, as well as the areas being positive for AP-2α mRNA, were separately microdissected and analysed. Finally, two AP-2α mRNA-positive paraffin-embedded samples were microdissected for reverse transcription polymerase chain reaction (RT-PCR) analyses.

### RT-PCR amplification

To study the expression of AP-2α mRNA, the total cellular RNA was extracted from five 20-μm-thick paraffin-embedded tissue sections of the tumour as described previously (Sugg et al, 1998). The RNA was used as a template to synthesize first-strand cDNA and PCR amplification was performed using the commercially available Enhanced Avian RT-PCR kit (Sigma, USA). The primers for the AP-2α were designed to anneal to exon 2 of the TFAP2A gene. The specific primers used were: AP-2 U 5′-GCCC-CGTGTCCCTGTCCAA-3′ and AP-2 L 5′-TGAGGAGCGA-GAGGCGACC-3′. PCR products were electrophoresed on a 6% polyacrylamide gel, stained with ethidium bromide and visualized under UV-light.

### LOH analysis

DNA was extracted by proteinase-K–phenol–chlorophorm method following standard protocols (Isola et al, 1994). Two TFAP2A-adjacent microsatellite markers, D6S470 (6.3 cM from TFAP2A) and D6S263 (7.9 cM from TFAP2A) were used to evaluate the frequency of LOH in AP-2α locus. These markers were chosen from the http://cedar.genetics.soton.ac.uk. Two other markers, D6S1027 and D6S1021, mapped to the long arm of chromosome 6 and were used to evaluate loss of the whole chromosome. Primer sequences and PCR amplification conditions to these markers were obtained from http://www.gdb.org. The forward primers were labelled either with fluorescent dyes 6-FAM or TET. The target sequences were amplified by PCR in a total volume of 20 μl with Ampli *Taq* Gold polymerase (Perkin-Elmer, Foster City, USA) following manufacturer's recommendations. Fragment analysis was done utilizing ABI PRISM 310 genetic analyser (Perkin-Elmer, Foster City, CA, USA).

LOH was calculated from the informative cases, i.e. where two alleles could be seen on the DNA obtained from normal tissue. Because PCR fragments of different sizes are amplified with different efficiences, the ratio of allele peak heights was calculated in matched normal and tumour DNA samples. The LOH-value was calculated using formula $LOH=(T2 \times N1)/(T1 \times N2)$, where T is a tumour allele and N a normal tissue allele. A value under 0.6 or greater than 1.67 was assessed LOH (Canzian et al, 1996).

### RESULTS

### AP-2α protein expression

In AP-2α immunohistochemistry, the normal epidermis stained positively throughout the series. In the specimens

© 2000 Cancer Research Campaign 

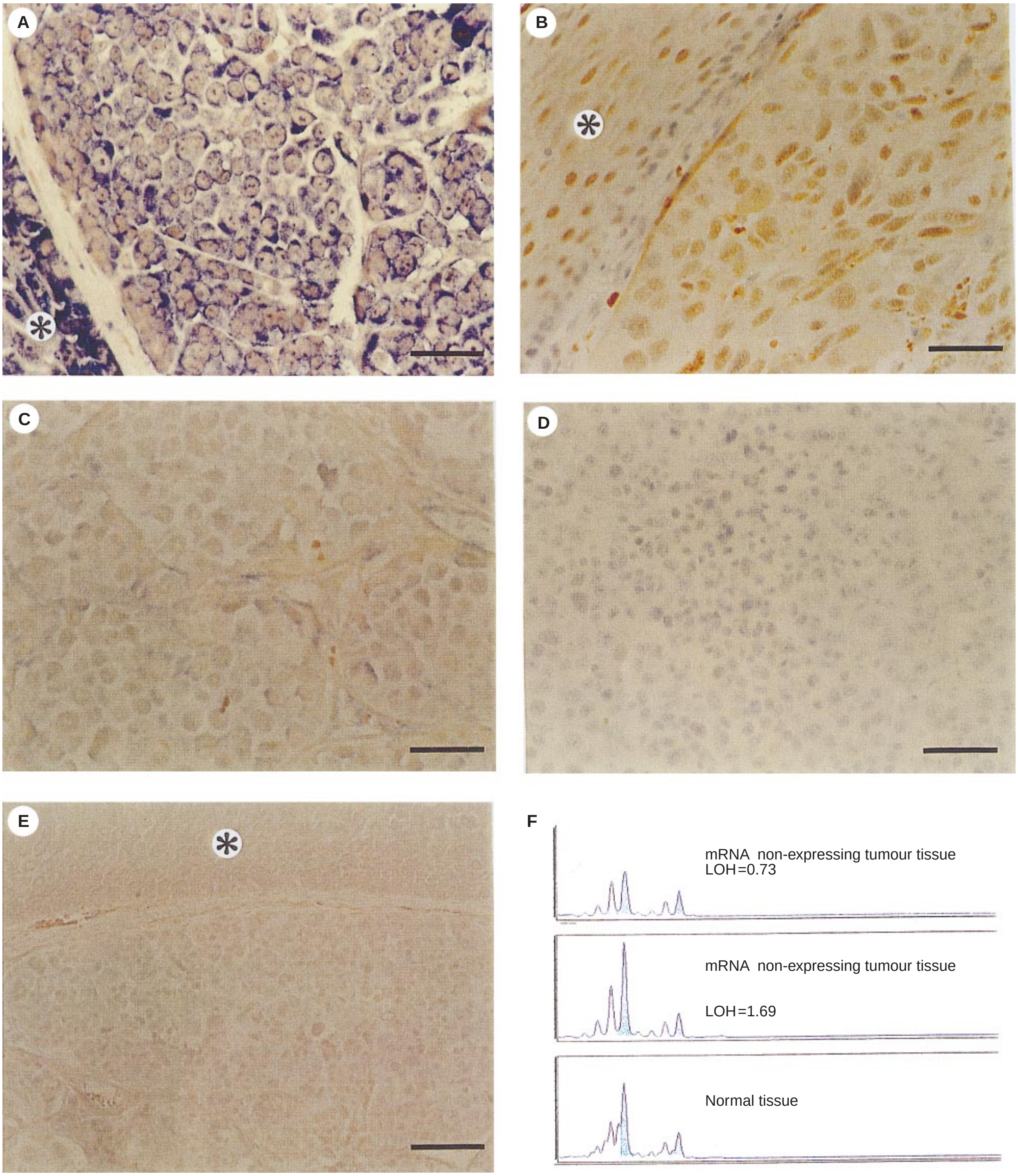

**Figure 1** AP-2α mRNA and protein expression as well as TFAP2A's LOH status in tumour areas of consecutive sections of the primary melanoma number 44. (**A, C, E**:) mRNA in situ hybridization; (**B, D**) immunohistochemistry. (**A**) mRNA-positive tumour area. Asterisk indicates normal epidermis. Bar = 40 μm. (**B**) Protein-positive tumour area. Asterisk indicates normal epidermis. Bar = 40 μm. (**C**) mRNA-negative tumour area. Bar = 40 μm. (**D**) Corresponding protein-negative tumour area as seen in **C**. Bar = 60 μm. (**E**) Corresponding tumour area to **A** stained using the sense probe (T7) as a negative control. Asterisk indicates normal epidermis. Bar = 80 μm. (**F**) Results of LOH analysis between the mRNA expressing (**A, B**) and mRNA non-expressing (**C, D**) tumour areas selected for the present study, 25 primary melanomas contained less than 75% and 27 primary melanomas contained more than 75% of positively stained tumour cells within a section.

### AP-2α mRNA expression

The mRNA signal was seen intracellularly as distinct blue dots (Figure 1A). The adjacent normal epidermis was constantly mRNA positive throughout the series. Of the 52 primary cutaneous

© 2000 Cancer Research Campaign

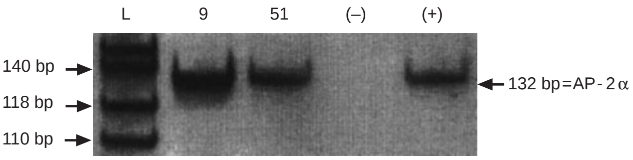

**Figure 2** Expression of AP-2α mRNA in cutaneous melanoma (cases 9 and 51). Exon 2 region of the TFAP2A gene was analysed by RT-PCR. The far left (L) line contains a size ladder indicating molecular weight, (–) depicts a negative control in which a template was replaced by water ($H_2O$), (+) is a positive control for AP-2α RNA (from Clontech Human Lung cDNA Library). Both cases (9, 51) contain PCR product of the same molecular size (132 bp) as the positive control sample

**Table 2** The comparisons of AP-2α mRNA and AP-2α protein expression patterns in consecutive sections of 52 primary cutaneous melanomas

| AP-2α protein expression | mRNA positive[a] *n* | mRNA negative[b] *n* | concordant mRNA- and protein expression patterns[c] *n* |
|---|---|---|---|
| < 75% (low) | 16 | – | 9 |
| ≥ 75% (high) | 24 | 2 | 1 |
| Total | 40 | 2 | 10 |

[a]mRNA-positive areas without concomitant protein expression. [b]Protein-positive areas without concomitant mRNA expression, cases 12 and 39. [c]Corresponding mRNA and protein-positive as well as -negative areas.

melanomas, 40 cases (77%) expressed mRNA throughout the section, and a focal staining pattern (containing also mRNA-negative areas) was observed in 12 (23%) cases (Table 2). RT-PCR from AP-2α mRNA- and protein-positive paraffin-embedded tumour tissues confirmed that the positive signal in ISH originated from the exon 2 of TFAP2A, instead of genomic sequences of AP-2β or artifacts in tissue processing (Figure 2).

### The relation of AP2-α mRNA to protein expression

In the subgroup of low AP-2α protein expression ($n$ = 25), 16 (64%) cases were completely mRNA-positive. Nine specimens (36%) displayed similar AP-2α protein and mRNA expression patterns, including concordantly mRNA- and protein-negative tumour areas. The highly AP-2α protein-positive tumours ($n$ = 27) were equally mRNA-positive in 25 (93%) cases. The two remaining specimens (7%) included AP-2α protein-positive areas without corresponding mRNA signal (Table 2).

### LOH analysis of TFAP2A

PCR-amplification was successful in a total of 17 primary cutaneous melanomas of which 13 (including 18 separately analysed tumour areas) were informative in the LOH analysis. LOH or loss of the chromosome 6 (monosomy) were found in 7/13 (54%) of all informative primary melanomas. Six out of seven of these chromosomal changes were found in tumours more than 1.5 mm in Breslow thickness (Table 1). There were five concordantly mRNA and protein negative areas in five tumours. These areas harboured LOH or monosomy in four cases (80%). On the other hand, all 13 tumours included mRNA-positive areas, which harboured two LOHs and three apparent monosomies (Figure 1F, Tables 1 and 3).

**Table 3** The findings of LOH analysis of TFAP2A according to AP-2α mRNA status in 18 analysed tumour areas in 13 primary cutaneous melanomas (only informative cases included)

| | Areas (*n*) | LOH[a] Yes (*n*) | LOH[a] No (*n*) |
|---|---|---|---|
| mRNA-positive | 13 | 5[b] | 8 |
| mRNA-negative | 5 | 4[c] | 1 |
| Total | 18 | 9 | 9 |

[a]Loss of heterozygosity. [b]Includes two cases with monosomy and one case in which monosomy could not be excluded. [c]Includes one case with monosomy and two cases in which monosomy could not be excluded.

### Metastatic lesions

Seven metastatic lesions were assessed for their AP-2α protein and mRNA status. The metastases had a smaller AP-2α protein-positive cell fraction than their corresponding primary tumours in four specimens. In one case the fractions of AP-2α protein-positive cells were equal, and in two cases the metastatic lesion had a higher number of AP-2α protein-positive cells than the corresponding primary tumour. The comparison of consecutive sections revealed mRNA without concomitant protein expression in four specimens. ISH and IHC sections were thoroughly positive in two specimens and one case displayed similar mRNA and protein expression patterns. This specimen had LOH only in the negative area.

### DISCUSSION

The present study showed that in the majority (64%) of the primary melanomas with reduced AP-2α protein expression the tumour tissue still contained AP-2α mRNA. This finding suggests that the AP-2α mRNA expression can be uncoupled from the AP-2α protein expression in primary cutaneous melanoma. Similarly, Butz et al (1998) found substantial discrepancies between intracellular p21 mRNA and protein levels in SW756 carcinoma cell lines under genotoxic stress. Our finding can be explained at least by two different ways. First, the AP-2α mRNA may not have been adequately translated into protein. This could be due to impaired post-transcriptional processing of the mRNA-transcript (Kleijn et al, 1998), or due to repression in translation initiation which is controlled by specific RNA–protein interactions (Day and Tuite, 1998; Kleijn et al, 1998). Also alternatively spliced AP-2α variant (Buettner et al, 1993) or specific translation inhibitory elements (Wang et al, 1997; Liu and Redmond, 1998) may function as translational repressors. Another possible reason for our result is premature truncation of the AP-2α protein which may affect the antibody affinity, or degradation of the AP-2α protein.

Because chromosomal deletions of a single allele copy (LOH) together with an inactivating point mutation in the remaining allele may result in loss of a proper gene product, and because LOHs may well point to possible tumour suppressor genes (Knudson, 1971; Baker et al, 1989; Levine and Momand, 1990; Sellers and Kaelin, 1997), we wanted to see whether the absence of AP-2α mRNA could be a consequence of LOH in the vicinity of TFAP2A. Ten primary cutaneous melanomas and one metastasis included well-matching protein and mRNA-negative areas, being potential candidates for LOH. Indeed, we found allelic loss in four out of five informative primary specimens and in the metastatic

© 2000 Cancer Research Campaign lesion, but we could not exclude loss of the whole chromosome in three of these cases with control markers mapping to 6q. Thus, LOH or monosomy of chromosome 6 seem to be quite frequent if the down-regulation of AP-2α protein is associated with the absence of AP-2α mRNA.

The genetic effect of chromosome 6 to melanoma was originally depicted by Trent et al with an observation that reintroduction of chromosome 6 into metastatic melanoma cells inhibited their tumourigenicity and metastatic potential (Trent et al, 1990). The long arm of the sixth chromosome (6q) is known to harbour LOH frequently in cutaneous melanoma (Millikin et al, 1991; Walker et al, 1994; Robertson et al, 1996; Slominski et al, 1998) and in other cancers (Foulkes et al, 1993; Noviello et al, 1996). So far, chromosomal losses confined specifically to the short arm of the chromosome 6 (6p) near the location of TFAP2A in 6p24 have been documented in melanoma cell lines (Real et al, 1998; Turner et al, 1998) and in several other malignancies (Foulkes et al, 1993). As far as the authors are aware, the present study is the first one to reveal LOHs confining specifically to 6p in primary cutaneous melanoma.

There were five LOHs (including three apparent monosomies) within the 13 informative mRNA-positive primary tumour areas (Table 3). This finding is similar to a previous observation of functional RB protein despite LOH of the *RB* locus (Dodson et al, 1994). Our findings suggest that the investigated chromosome region around TFAP2A locus or more distant regions in chromosome 6 may contain other putative tumour suppressor genes that have a role in melanoma progression. There were three cases (numbers 12, 39 and 40) with unexpected results in the analyses. The discrepancy between mRNA and protein expression in cases 12 and 39 could theoretically be explained by prolonged protein's half-life, autoregulation mechanisms suppressing AP-2α's mRNA expression (Imhof et al, 1999) or alternative mRNA splicing affecting the mRNA ISH probe affinity (Meier et al, 1995; Ohtaka-Maruyama et al, 1998). It is also possible that the absence of AP-2α mRNA in the case number 40 is due to other genetic inactivation mechanisms than LOH, such as gene methylation (Gonzalgo et al, 1997) or mutations affecting both gene alleles (Levine and Momand, 1990; Sellers and Kaelin, 1997). Being rare these three cases do not influence the main conclusions of the current study, but the possible role of these mechanisms should be studied in future.

To further evaluate the role of other AP-2 family proteins (Turner et al, 1998) in the present series, we tested primary cutaneous melanoma specimens with high and low AP-2α protein expression for their AP-2β and AP-2γ expression. Despite a weak cytoplasmic signal with both antibodies in less than half of the cases, none of the tumours displayed any nuclear positivity (data not shown). The AP-2α-specific RT-PCR further confirmed that the positive mRNA signal seen with ISH originated from AP-2α. Supporting our finding, AP-2α has also been demonstrated to be the principal AP-2 form in keratinocytes by Northern blots (Bossler AD et al, manuscript in preparation). Thus, it seems that the immunopositivity detected by C-18 antibody represents mainly AP-2α, and that AP-2α is the most important AP-2 gene family member related to the progression of skin melanoma.

Our results suggest that the expression of AP-2α protein in melanoma is mainly under post-transcriptional control, and the absence of mRNA seems to comprise the remaining one-third of the clinically significant down-regulation. When mRNA was not detected, specific LOH for the 6p arm or monosomy was frequently (80%) encountered. According to our results, LOH within TFAP2A-adjacent area is a late phenomenon in melanoma progression. The finding of AP-2α mRNA and protein despite simultaneous LOH suggests that there may be a new putative tumour suppressor gene within close vicinity of TFAP2A. In addition to these novel findings our study has opened many questions which need to be answered in future studies to understand more precisely the role of AP-2α in cutaneous melanoma.

### ACKNOWLEDGEMENTS

This study was supported by grants from the Kuopio University Foundation, the Cancer Fund of Finland and by EVO-funding of Kuopio University Hospital. The authors thank Ms Seija Haatanen, Ms Aija Parkkinen, Ms Helena Kemiläinen, Ms Irma Väänänen, Ms Raija Pitkänen, Mr Alpo Pelttari and Ms Eeva Sallinen for their skilful technical assistance.

### REFERENCES

Baker SJ, Fearon ER, Nigro JM, Hamilton SR, Preisinger AC, Jessup JM, van Tuinen P, Ledbetter DH, Barker DF and Nakamura Y (1989) Chromosome 17 deletions and p53 gene mutations in colorectal carcinomas. *Science* **244**: 217–221

Bar-Eli M (1997) Molecular mechanisms of melanoma metastasis. *J Cell Physiol* **173**: 275–278

Bosher JM, Williams T and Hurst HC (1995) The developmentally regulated transcription factor AP-2 is involved in c-erbB-2 overexpression in human mammary carcinoma. *Proc Natl Acad Sci USA* **92**: 744–747

Buettner R, Kannan P, Imhof A, Bauer R, Yim SO, Glockshuber R, Van Dyke MW and Tainsky MA (1993) An alternatively spliced mRNA from the AP-2 gene encodes a negative regulator of transcriptional activation by AP-2. *Mol Cell Biol* **13**: 4174–4185

Butz K, Geisen C, Ullman A, Zentgraf H and Hoppe-Seyler F (1998) Uncoupling of $p21^{WAF1/CIP1/SDI1}$ mRNA and protein expression upon genotoxic stress. *Oncogene* **17**: 781–787

Canzian F, Salovaara R, Hemminki A, Cristo P, Chadwich RB, Aaltonen LA and de-la-Chapelle A (1996) Semiautomated assessment of loss of heterozygosity and replication error in tumors. *Cancer Res* **56**: 3331–3337

Day DA and Tuite MF (1998) Post-transcriptional gene regulatory mechanisms in eukaryocytes: an overview. *J Endocrinol* **157**: 361–371

Dodson MK, Cliby WA, Xu HJ, DeLacey KA, Hu SX, Keeney GL, Li J, Podratz KL, Jenkins RB and Benedict WF (1994) Evidence of functional RB protein in epithelial ovarian carcinomas despite the loss of heterozygosity at RB locus. *Cancer Res* **54**: 610–613

Foulkes WD, Ragoussis J, Stamp GWH, Allan GJ and Trowsdale J (1993) Frequent loss of heterozygosity on chromosome 6 in human ovarian carcinoma. *Br J Cancer* **67**: 551–559

Gaynor RB, Muchard C, Xia Y, Klisak I, Mohandas T, Sparkes RS and Lusis AJ (1991) Localization of the gene for the DNA-binding protein AP-2 to human chromosome 6p22.3-pter. *Genomics* **10**: 1100–1102

Gonzalgo ML, Bender CM, You EH, Glendening JM, Flores JF, Walker GJ, Hayward NK, Jones PA and Fountain JW (1997) Low frequency of p16/CDKN2A methylation in sporadic melanoma: comparative approaches for methylation analysis of primary tumors. *Cancer Res* **57**: 5336–5347

Huang S, Jean D, Luca M, Tainsky MA and Bar-Eli M (1998) Loss of AP-2 results in downregulation of c-KIT and enhancement of melanoma tumorigenicity and metastasis. *EMBO J* **17**: 4358–4369

Imhof A, Schuierer M, Werner O, Moser M, Roth C, Bauer R and Buettner R (1999) Transcriptional regulation of the AP-2alpha promoter by BTEB-1 and AP-2rep, a novel wt-1/egr-related zinc finger repressor. *Mol Cell Biol* **19**: 194–204

Isola J, De Vries S, Chu L, Chazvini S and Waldman F (1994) Analysis of changes in DNA sequence copy number by comparative genomic hybridization in archival paraffin-embedded tumor samples. *Am J Pathol* **115**: 1301–1308

Jean D, Gershenwald JE, Huang S, Luca M, Hudson MJ, Tainsky MA and Bar-Eli M (1998) Loss of AP-2 results in up-regulation of MCAM/MUC18 and an increase in tumor growth and metastasis of human melanoma cells. *J Biol Chem* **273**: 16501–16508

 © 2000 Cancer Research Campaign

Karjalainen JM, Kellokoski JK, Eskelinen MJ, Alhava EM and Kosma VM (1998) Downregulation of transcription factor AP-2 predicts poor survival in stage I cutaneous malignant melanoma. *J Clin Oncol* **16**: 3584–3591

Kleijn M, Scheper GC, Voorma HO and Thomas AAM (1998) Regulation of translation initiation factors by signal transduction. *Eur J Biochem* **253**: 531–544

Knudson AGJ (1971) Mutation and cancer: statistical study of retinoblastoma. *Proc Natl Acad Sci USA* **68**: 820–823

Levine AJ and Momand J (1990) Tumor suppressor genes: the p53 and retinioblastoma sensitivity genes and gene products. *Biochimica Biophysica Acta* **1032**: 119–136

Liu SY and Redmond TM (1998) Role of the 3′-untranslated region of RPE65 mRNA in the translational regulation of the RPE65 gene: identification of a specific translation inhibitory element. *Arch Biochem Biophys* **357**: 37–44

Meier P, Koedood M, Philipp J, Fontana A and Mitchell PJ (1995) Alternative mRNAs encode multiple isoforms of transcription factor AP-2 during murine embryogenesis. *Dev Biol* **169**: 1–14

Millikin D, Meese E, Vogelstein B, Witkowski C and Trent J (1991) Loss of heterozygosity for loci on the long arm of chromosome 6 in human malignant melanoma. *Cancer Res* **51**: 5449–5453

Mitchell PJ, Wang C and Tjian R (1987) Positive and negative regulation of transcription in vitro: enhancer-binding protein AP-2 is inhibited by SV40 T antigen. *Cell* **50**: 847–861

Mitchell PJ, Timmons PM, Hebert JM, Rigby PW and Tjian R (1991) Transcription factor AP-2 is expressed in neural crest cell lineages during mouse embryogenesis. *Genes Dev* **5**: 105–119

Noviello C, Courjal F and Theillet C (1996) Loss of heterozygosity on the long arm of chromosome 6 in breast cancer: possibly four regions of deletion. *Clin Cancer Res* **2**: 1601–1606

Ohtaka-Maruyama C, Hanaoka F and Chepelinsky AB (1998) A novel alternative spliced variant of the transcription factor AP2alpha is expressed in the murine ocular lens. *Dev Biol* **202**: 125–135

Real LM, Jimenez P, Canton J, Kirkin A, Garcia A, Abril E, Zeuthen J, Ruiz-Cabello F and Garrido F (1998) In vivo and in vitro generation of a new altered HLA phenotype in melanoma-tumour-cell variants expressing a single HLA-class-I allele. *Int J Cancer* **75**: 317–323

Robertson GP, Coleman AB and Lugo TG (1996) Mechanisms of human melanoma cell growth and tumor suppression by chromosome 6. *Cancer Res* **56**: 1635–1641

Sellers WR and Kaelin WGJ (1997) Role of the retinoblastoma protein in the pathogenesis of human cancer. *J Clin Oncol* **15**: 3301–3312

Slominski A, Wortsman J, Nickoloff B, McClatchey K, Mihm MC and Ross JS (1998) Molecular pathology of malignant melanoma. *Am J Clin Pathol* **110**: 788–794

Sugg SL, Ezzat S, Rosen IB, Freeman JL and Asa SL (1998) Distinct multiple RET/PTC gene rearrangements in multifocal papillary thyroid neoplasia. *J Clin Endocrinol Metabol* **83**: 4116–4122

Trent JM, Stanbridge EJ, McBride HL, Meese EU, Casey G, Araujo D, Witkowski CM and Nagle RB (1990) Tumorigenicity in human melanoma cell lines controlled by introduction of human chromosome 6. *Science* **247**: 568–571

Turner BC, Zhang J, Gumbs AA, Maher MG, Kaplan L, Carter D, Glazer PM, Hurst HC, Haffty BG and Williams T (1998) Expression of AP-2 transcription factors in human breast cancer correlates with the regulation of multiple growth factor signalling pathways. *Cancer Res* **58**: 5466–5472

Walker GJ, Palmer JM, Walters MK, Nancarrow DJ, Parsons PG and Hayward NK (1994) Simple tandem repeat allelic deletions confirm the preferential loss of distal chromosome 6q in melanoma. *Int J Cancer* **58**: 203–206

Wang E, Ma W-J, Aghajanian C and Spriggs DR (1997) Posttranscriptional regulation of protein expression in human epithelial carcinoma cells by adenine-uridine-rich elements in the 3′-untranslated region of tumor necrosis factor-α messenger RNA. *Cancer Res* **57**: 5426–5433

Williamson JA, Bosher JM, Skinner A, Sheer D, Williams T and Hurst HC (1996) Chromosomal mapping of the human and mouse homologues of two new members of the AP-2 family of transcription factors. *Genomics* **35**: 262–264

Zeng YX, Somasundaram K and el-Deiry WS (1997) AP2 inhibits cancer cell growth and activates p21 WAF1/CIP1 expression. *Nat Genet* **15**: 78–82

*© 2000 Cancer Research Campaign* 
